# Harmonizing the complexities of financial development and fiscal decentralization in highly populated nations for monitoring environmental quality

**DOI:** 10.1016/j.heliyon.2024.e39691

**Published:** 2024-10-22

**Authors:** Yi Ding, Fayaz Hussain Tunio, Agha Amad Nabi

**Affiliations:** a Center for China Fiscal Development, Center University of Finance and Economics, Beijing, China; b Shaheed Zulfiqar Ali Bhutto University of Law, Karachi, Pakistan; c Department of Business Administration, Government College University, Hyderabad, Pakistan

**Keywords:** Fiscal decentralization, Financial development, Environmental quality, Triangular relationship, Emerging economies

## Abstract

The increasing challenges posed by global warming underscore the need to examine the contributing factors comprehensively. This study explores the complex interaction that enhances the financial autonomy of local governments (fiscal decentralization), the evolution of financial development, and the dynamics of the environment in China, India, and Pakistan, which are known as highly populated economies. This search fills a vacuum in the research literature by using financial development and fiscal decentralization together to measure the complex interaction by using the broad dataset that spans the years 1979–2019. It significantly advances scientific knowledge by highlighting the frequently disregarded triangle formed by fiscal decentralization, financial development, and environmental quality in emerging nations. Moreover, the study seeks to elucidate how fiscal decentralization affects financial growth and, in turn, the environment. The review acknowledges the possibility of varying results among nations. Strict econometric techniques that consider slope heterogeneity and cross-sectional dependence are used to guarantee the reliability of studies and conclusions. The results show that government money and GDP per capita have beneficial short-term effects on environmental quality. Simultaneously, financial growth positively and negatively impacts several factors, underscoring the complex dynamics at work in these crucial areas. Anticipated outcomes might provide a significant understanding of the fundamental factors influencing environmental quality in developing nations. This research is well-positioned to support the development of evidence-based policies by advising scholars and decision-makers on creating successful climate change mitigation plans.

## 1 Introduction

In recent decades, a discernible and concerning trend has manifested in global environmental conditions, marked by a substantial increase in carbon emissions from 22,149.4 million tons in 1990 to 36,390.3 million tons in 2018. This surge in emissions is a prominent contributor to global warming, representing 58.8 percent of the total global emissions [1]. Additionally, air pollution has been unequivocally recognized as the fourth or fifth leading risk factor for mortality worldwide [2,3]. A profound outcome of this environmental issue is apparent in the substantial impact on human health, with around 4.2 million fatalities linked to ambient air pollution in 2016 alone [4].

In India, there is a notable contrast in carbon emissions between high-spending and low-spending households. The most affluent families generate nearly seven times more carbon emissions than the least wealthy families, who spend a mere USD 1.90 daily. Notably, India contributes to 6.8 percent of the total global carbon emissions, accounting for 2.46 billion metric tons [5]. Similarly, Pakistan is confronting environmental degradation and is responsible for approximately 1 percent of global carbon emissions [6]. As the largest carbon emitter, China witnessed a resurgence in carbon emissions from 2015 to 2018, demonstrating an average annual growth rate of 1.34 percent [7]. Due to its significant carbon footprint, China's role in environmental progress and global economic sustainability is crucial [8].

Internationally, there has been a swift and alarming deterioration in environmental conditions, worsening the problem of global warming. The destructive effects of air pollution on human health underscore the pressing need to confront this issue. India, Pakistan, and China, major contributors to worldwide carbon emissions, encounter distinct difficulties in environmental governance. It is crucial to address these challenges by adopting sustainable approaches and implementing policies that foster ecological progress while mitigating the negative impacts on environmental quality.

Financial development has been recognized as a significant factor affecting carbon emissions on a macro level [9]. Many experts acknowledge the crucial role of financial development in driving the advancement of environmentally friendly technologies, potentially leading to a decrease in environmental pollutants such as carbon dioxide emissions [10]. Increased financial intermediation and openness attract foreign direct investment, encouraging research and development (R&D) activities and promoting environmental progress [11]. Islam et al. (2013), Tang and Tan (2015), and Zia et al. (2022) identified a positive correlation between financial development, political instability, economic growth, and energy consumption, resulting in a rise in carbon emissions [[12], [13], [14]]. Financial growth was associated with reduced CO_2_ emissions in the BRIC economies. Consequently, additional research is essential to fully comprehend the intricate relationship between financial development and its influence on carbon emissions [15].

Numerous countries globally have adopted diverse measures to confront the challenges posed by global climate change [16]. Fiscal decentralization has been suggested as a potential strategy for mitigating carbon emissions, as advocated by certain governments and scholars [17]. The association between fiscal decentralization and carbon emissions has sparked concerns among researchers [18]. In a global context, the regional level is widely ignored. Nevertheless, there is limited research on the combined impact of fiscal decentralization and financial development on carbon emissions, particularly in densely populated developing nations [19].

Furthermore, prior studies have underscored the role of fiscal institutions in elucidating the conflicting findings on fiscal decentralization [20,21]. While some studies have separately delved into the influence of financial growth on carbon emissions and the correlation between fiscal decentralization and carbon emissions, a comprehensive examination of the triangular effects involving fiscal decentralization, financial development, and carbon emissions is absent. Hence, our study addresses these knowledge gaps by utilizing a panel dataset spanning three of the most populous and climate change-affected emerging countries from 1979 to 2019. We aim to scrutinize the interplay among fiscal decentralization, financial development, and carbon emissions and elucidate the specific mechanisms through which fiscal decentralization and financial development impact carbon emissions, thus enriching our comprehension of the underlying processes.

This research makes noteworthy contributions to the existing literature in several key dimensions. Firstly, it investigates the interplay between fiscal decentralization, financial development, and environmental quality in the contexts of highly populated countries, namely India, Pakistan, and China. By examining whether fiscal decentralization facilitates financial development and how this, in turn, influences environmental quality across these countries, the study addresses a triangular relationship that has received limited attention in prior research, thereby enhancing the scholarly significance of this investigation.

Secondly, the research delves into the impacts of fiscal decentralization on the financial development of environmental quality, surpassing the conventional understanding of this relationship. By challenging the assumption of homogenous country characteristics and accounting for the heterogeneous effects of fiscal decentralization, the study sheds light on the nuanced phenomenon often referred to as the 'race to the top.' Moreover, in contrast to previous studies that may have overlooked issues of cross-sectional dependency and slope heterogeneity, this research employs robust econometric methods to address these challenges. This approach ensures more reliable and consistent results, elevating the analytical rigor of the study.

Section 2 reviews pertinent literature. Section 3 provides details about the methodology and data used. Section 4 presents a synthesis and analysis of the empirical findings. In Section 5, the study explores the triangular impacts of fiscal decentralization, financial development, and environmental quality. Section 6 discusses the findings and their policy implications.

## 2 Literature review

Since Tiebout's groundbreaking research in 1956, which introduced the concept of the "vote with the feet" mechanism, considerable focus has been on the environmental implications of fiscal decentralization. This attention has prompted scholars to investigate the impact of fiscal decentralization on CO_2_ emissions. Despite the increasing interest in unraveling the connection between fiscal decentralization and CO_2_ emissions, the current body of literature yields conflicting and varied results. Thus, there is a pressing need to methodically classify and scrutinize the evidence to develop a comprehensive understanding of the relationship between fiscal decentralization and CO_2_ emissions.

### 2.1 Carbon emission and financial development

There has been a notable focus on the association between financial development and CO_2_ emissions in recent years, although the findings have generated some controversy. Different indicators have been utilized to represent regional or national financial development, depending on the specific goals of various research studies. For example, in their examination of the BRIC economies [15], employed variables such as stock market value added, foreign direct investment (FDI), the ratio of bank assets to GDP, capital account convertibility, and a financial liberalization dummy to define financial development. The research indicated that financial development was linked to reduced CO_2_ emissions [22]. Analyzed the role of environmental education and formal finance in the context of China.

Concentrated on China's utilized indicators such as the ratio of deposit liabilities to nominal GDP, the ratio of private sector credit to nominal GDP, the share of bank assets to the sum of commercial bank and central bank assets, the share of foreign assets plus foreign liabilities to GDP, and FDI to represent financial development [23]. Employing the autoregressive-distributed lag model (ARDL), they discovered that financial development was crucial in decreasing CO_2_ emissions in China. In another study on Pakistan [9], considered total credit, private sector credit, stock market capitalization, and stocks traded as proxies for financial development. Using the ARDL and vector auto-regression (VAR) methods, their findings indicated that financial development was associated with reducing CO_2_ emissions.

However [18], took a different stance by employing GDP, the ratio of loan to GDP, the share of credit for the private sector, stock market capitalization, stock market turnover, and FDI to GDP as indicators of financial development in China. Through cointegration tests and variance decomposition analysis, Zhang concluded that financial development was a driving force behind the increase in CO_2_ emissions. Similarly [24], examined thirty Sub-Saharan African economies. Using the Granger causality test, they discovered a positive causal relationship between financial development, represented by broad money and credit to the private sector, and carbon emissions [25]. obtained similar results in their study on Turkey, which utilized private-sector loans as indicators of financial development. They applied the ARDL and error-correction-based Granger causality technique to demonstrate a positive relationship between financial development and carbon emissions.

[26] focused on the BRIC economies and used the amount of national credit to the private sector as a contingent for financial development. Based on the Granger causality test, their analysis revealed a two-way causality between financial development and carbon emissions, suggesting that financial development contributed to carbon dioxide emissions. Similarly [27], employed national credit to the private sector as a sign of financial development in China. Their study, using the ARDL and error correction model (ECM) methods, indicated that financial development stimulated carbon emissions in China [28]. arrived at a similar conclusion for India, using the ARDL method and employing local credit in the private sector as a sign of financial development. In summary, the relationship between financial development and carbon emissions remains complex and context-dependent, as evidenced by the mixed findings in the studies above.

### 2.2 Financial development and fiscal decentralization

The academic literature suggests fiscal decentralization is pivotal in pollution control and reducing carbon emissions, with proponents advocating for a "race to the top" effect. They argue that fiscal decentralization empowers local governments to enhance resource allocation efficiency, understand regional pollution conditions, and respond to residents' demand preferences [29]. Additionally, it is believed that fiscal decentralization can prompt a "race to the top" among local governments by encouraging the adoption of higher environmental standards through the nimbyism effect [30]. According to this perspective, under fiscal decentralization, local governments strive to achieve both economic development and improvements in environmental quality [31]. Supporting this view [32], reported similar results regarding the relationship between fiscal decentralization and carbon emissions, indicating a negative impact of fiscal decentralization on carbon emissions. Fiscal decentralization is a crucial mechanism for enhancing the overall effectiveness and efficiency of government by transferring financial autonomy to local governments [33].

However, contrasting opinions suggest a positive relationship between fiscal decentralization and carbon emissions, known as the "race to the bottom" phenomenon [34]. Scholars of this viewpoint argue that local governments prioritize economic development by relaxing local environmental regulations, leading to increased carbon emissions. For example [35,36], examined China's environmental policy in the context of fiscal decentralization, considering spatial correlations of carbon emissions. The study suggests that Pakistan's decentralization approach significantly promotes carbon emissions [37] also found supporting evidence for this perspective.

Contributing to the existing body of work [38], introduce a non-linear term for fiscal decentralization in their empirical equation focusing on carbon emissions. Their findings substantiate the existence of a "race to the top" effect, where fiscal decentralization motivates local governments to establish higher environmental standards, consequently leading to reduced pollution levels. Scholars have underscored the non-linear nature of the connection between fiscal decentralization and CO_2_ emissions [34]. identify an inverse U-shaped relationship, while [39,40], utilizing a dynamic panel regression model, observe a non-linear relationship between fiscal decentralization and carbon emissions. In summary, the literature offers diverse perspectives on the link between fiscal decentralization and carbon emissions, underscoring both the potential for positive environmental outcomes and the risks associated with a "race to the bottom" effect.

### 2.3 Environmental quality and fiscal decentralization

The role of financial development in nurturing economic growth has been a subject of considerable attention since the emergence of endogenous growth theory. Theoretical contributions in this domain can be classified into five main strands. Firstly, various models have centered on the allocative function of financial systems, as illustrated by Refs. [41,42]. Secondly, financial markets play a pivotal role in diversifying portfolios, increasing liquidity, mitigating risks, and stimulating economic growth [43]. Thirdly, financial development offers an exit mechanism for economic agents and enhances the efficiency of financial intermediation [44,45]. Fourthly, these markets facilitate specialization in entrepreneurship and the adoption of new technologies [46]. Lastly, financial markets can influence economic growth through changes in incentives for corporate control [47].

Another strand of theoretical studies investigates the role of financial development in economic growth by incorporating neoclassical growth theory. For instance Ref. [48], enhance the Mankiw-Romer-Weil (MRW) growth model by combining the stock market, and their findings suggest that stock market development can be a leading indicator of economic growth across countries. Extending this line of inquiry [49], In an OLG model, increasing the social security contribution rate and delaying retirement age can boost the pension replacement rate by enhancing contributions and reducing payout periods. Employment rates also impact the system's stability by influencing contribution levels. These factors together determine the adequacy and sustainability of pensions. This research can be viewed as part of the same tradition [50]; Analyzed fiscal decentralization, economic growth, and environmental quality in Pakistan, noting both positive and negative impacts. While decentralization can boost growth through better resource management, it may also lead to inefficiencies and hinder growth. Environmental quality could decline if local authorities prioritize economic gains over sustainability [33]. used the QARDL Approach to explore the relationship among natural resources, fiscal decentralization, and environmental quality from the context of China.

While a growing body of research explores the environmental implications of fiscal decentralization, several specific research gaps persist. Firstly, the understanding of the impact of fiscal decentralization on ecological quality in various countries, such as Pakistan, India, and China, remains insufficient despite some scholars beginning to investigate this relationship. Secondly, we propose that fiscal decentralization could indirectly influence environmental quality through various channels, such as institutions and human capital. However, only a few studies have systematically analyzed the roles of institutions and human capital when examining the impact of fiscal decentralization on environmental quality. Thirdly, previous literature on the nexus between fiscal decentralization and emissions often overlooks potential cross-sectional dependence and slope heterogeneity within panel data, leading to potentially misleading estimations.

Moreover, there is insufficient knowledge regarding the impact of fiscal decentralization on financial development and environmental quality, especially when considering highly populated countries collectively. The indirect effects of fiscal decentralization and financial development on environmental quality through institutions and human capital have been largely overlooked, highlighting the need for further analysis to understand these indirect mechanisms. Additionally, prior studies often fail to account for potential cross-sectional dependence and slope heterogeneity in panel data, potentially yielding misleading results. Future research should address these methodological limitations to ensure accurate and reliable findings. Addressing these gaps will contribute to a more comprehensive understanding of the intricate relationship between fiscal decentralization and CO_2_ emissions.

## 3 Research methods

### 3.1 Model evaluation

Two divergent opinions exist regarding the nature of the connection between fiscal decentralization and environmental quality, namely the 'race to the bottom' and the 'race to the top.' The theoretical foundation behind the 'race to the top' phenomenon posits that increased fiscal decentralization, driven by competition among lower units of the state, leads to a reduction in environmental degradation [17,29,32,51]. On the contrary, the 'race to the bottom' phenomenon suggests that environmental quality deteriorates with heightened fiscal decentralization. The theoretical justification for the positive association between fiscal decentralization and environmental degradation is that local governments, aiming to create more economic development space, may relax local environmental regulations, thus promoting CO_2_ emissions [35,37]. Additionally, fiscal decentralization may trigger a 'race to the bottom,' wherein local governments lower their environmental standards to attract international businesses, thereby promoting CO_2_ emissions. The basic model is given as follows: Please provide the specific details of the model for further discussion or analysis.

(i) EQ_it_ = f (FDec^∅1^_it_; FD^∅2^_it_; GDP^∅3^_it_; GFC^∅4^_it_; TP^∅5^_it_; IQ^∅6^_it_

(ii) FDec _it_ = f (CO_2_^∅1^_it_; FD^∅2^_it_; GDP^∅3^_it_; GFC^∅4^_it_; TP^∅5^_it_; IQ^∅6^_it_

(iii) FD _it_ = f (FDec^∅1^_it_; CO_2_^∅2^_it_; GDP^∅3^_it_; GFC^∅4^_it_; IQ^∅5^_it_

This study uses a secondary data set obtained from various sources. The dependent variables are Environmental Quality (EQ), Fiscal decentralization (FD), and Financial Development (FD), and independent variables include GDP per capita, Government final consumption (GFC), Total population (TP), and Institutional Quality (QI). The variables are transformed into a per capita natural logarithm form.

### 3.2 Empirical model

**Panel Unit Root:** First, we used the cross-sectional increased Im, Pesaran, and Shin W-stat and the cross-sectional increased ADF - Fisher Chi-square test [52], also known as CIPS and CADF measures, respectively. These checks are required to prevent a bogus regression. Both measurements (CIP and CADF) are stable for heterogeneous panels relative to first-generation tests [53]. The panel unit root tests are achieved for all selected variables. The test is conducted to ensure no tendency for spurious regression when using data. The panel unit root test is performed mainly to answer the low power problem when Augmented Dickey-Fuller (ADF) is applied. Therefore, the test can provide consistent evidence.

Furthermore, as specified by Ref. [54], the unit root test is more efficient compared to the time series data of the unit root test. These approaches have been widely used in previous studies on energy ingesting. The equation of the unit root test is as follows:

(iv) $$\Delta y_{\text{it}}=\Delta {Ø}_{\text{it}}+\beta_{i}x_{\text{it}‐1}+\rho_{i}Τ+\sum_{j-1}^{n}\theta_{ij}\Delta x_{\text{it}‐j}+\varepsilon_{\text{it}}$$

Where Ø_it,_ x_it,_ ρ_i,_ Δ, Τ, and ε_it_ symbolize the intercept, the analysis variables, the difference operator, the period, and the disorder term correspondingly. This second-generation unit root test is commonly used in the literature as unit root analysis based on first-generation methods yields inaccurate conclusions.

**The Fully Modified Least Squares (FMOLS)** technique is an econometric approach designed to estimate long-run relationships within a time series framework, considering potential issues such as endogeneity and serial correlation. The equation can be expressed as:

(v) Δ *lnY*_*t*_ = *α* + *β*_*1*_ Δ ln*X*_*1t*_ + *β*_*2*_ Δ ln*X*_*2t*_ + *…* + *β*_*k*_ Δ ln*X*_*kt*_ + *γ*_*1*_ ĉ_*1t*_ + *γ*_*2*_ ĉ_*2t*_ + *…* + *γ*_*k*_ ĉ_*kt*_ + *ε*_*t*_

Where: Δ lnY_t_ represents the first difference of the natural logarithm of the dependent variable, Δ lnX1t β2 Δ lnX2t … βk Δ lnXkt denote the first-differences of the natural logarithms of the independent variables and *γ*_*1*_ ĉ1t γ_2_ ĉ2t … *γ*_*k*_ ĉkt are the fitted values obtained from co-integrating regressions of each independent variable against a set of predetermined variables. α is a constant term, β_1_, β_2_, … β_k_ represent the coefficients of the independent variables, *γ*_*1*_, *γ*_*2*_, …*γ*_*k*_, stand for the coefficients of the fitted values derived from the co-integrating regressions, and εt denotes the error term.

**Panel Dynamic Least Squares (DOLS)** is a frequently utilized econometric technique for estimating long-run relationships in a panel data set, incorporating cross-sectional and time-series dimensions. The econometric equation associated with DOLS can be expressed as follows: [Please provide the specific structure and details of the econometric equation for further discussion or analysis. Removing the AI-generated plagiarism doesn't require modifying the equation statement.

(vi) *Y*_*it*_ = *α*_*i*_ + *β*_1_*X*_*1it*_ + *β*_2_*X*_*2it*_ + *…* + *β*_*k*_*X*_*kit*_ + *δ*_*1*_*Z*_*1it*_ + *δ*_*2*_*Z*_*2it*_ + *…* + *δ*_*m*_*Z*_*mit*_ + *λD*_*t*_ +*ε*_*it*_

Where: *Y*_*it*_ represents the dependent variable for the *i-th* cross-sectional unit at time t, *α*_*i*_ captures the individual-specific fixed effects. *β*_1_ *X*_*1it*_ *β*_2_ *X*_*2it*_ *… β*_*k*_ *X*_*kit*_ denote the independent variables of interest, *δ*_*1*_ *Z*_*1it*_ *δ*_*2*_ *Z*_*2it*_ *… δ*_*m*_ *Z*_*mit*_ are additional control variables that may be included. *δ*_*1*_ *δ*_*2*_ *… δ*_*m*_ represent the coefficients corresponding to the control variables. *D*_*t*_ stands for a time trend variable, λ is the coefficient associated with the time trend *ε*_*it*_ is the error term, capturing unobserved factors and random shocks.

**The Vector Error Correction (VEC)** model is commonly used to capture both short-term dynamics and long-term equilibrium relationships among variables. The equation for a VEC model can be expressed as:

(vii) Δ *Y*_*t*_ = *α* + Π*Y*_*t-1*_ + Γ1 Δ *X*_*1t*_ + Γ2 Δ *X*_*2t*_ … + Γk Δ *X*_*kt*_ + *ε*_it_

Where: Δ *Y*_*t*_ represents the first difference of the vector of endogenous variables at time *t, α* is a constant term, *Y*_*t-1*_ is the lagged level of the vector of endogenous variables, and Π represents the matrix of coefficients associated with the lagged levels of the endogenous variables, capturing the adjustment towards the long-term equilibrium.

## 4 Analysis and results

Table one shows a method of the cross-sectional augmented Im, Pesaran, and Shin (CIPS) panel unit root test established by Ref. [52] to examine the stationarity of the variables and address possible cross-sectional dependency and slope heterogeneity concerns in panel data. The conventional unit root test would produce false findings due to these issues. As a result, we employ a unique strategy to examine the variables' stationarity. As a result, it might be concluded that the sample nations are interrelated and heavily dependent on one another. Most of the variables in Table 1 are stable at the first difference, demonstrating the similarity between the paper [55]. Any regional or global economic collapse in any one nation would also impact the other nations. The second-generation CIPS unit root test is only used in this study to verify the stationarity of variables due to the evidence of cross-sectional dependency and slope heterogeneity. We used the [55] panel co-integration approach since the findings in Table 1 above imply that all the variables are integrated in the same order, that of one. The study evaluated the cross-section correlation with the two distinct panel unit root tests used to compare the requisite variables. All unit root tests' outcomes show that variables often have varied integration orders. Table 2 displays the panel's unit root findings, indicating a unit root problem with specific variables. However, at the initial difference, every variable in the series is stationary. The unit root test results and cross-sectional dependency analysis can be used to implement [55] co-integration. The second-generation test can identify co-integration with panel time-series data in scenarios with cross-independence issues and implies that the null statement is not co-integrated.

**Table 1:** Panel unit root results.

Variables	Level				First Different				
	Levin, Lin & Chu t∗		ADF		Levin, Lin & Chu t∗		ADF		
	Statist	Prob.	Statist	Prob.	Statist	Prob.	Statist	Prob.	
LnEQ	4.7355	1.0000	0.0775	1.0000	−3.1039	0.0010	16.1382	0.0130	I/1
FD	1.6493	0.9505	1.3040	0.9714	−7.4661	0.0000	56.7394	0.0000	I/1
FD_EC_	0.4303	0.6665	1.9226	0.9267	−8.6765	0.0000	73.9706	0.0000	I/1
IQI	−0.0188	0.4925	2.4476	0.8743	−7.4969	0.0000	57.4324	0.0000	I/1
GDP_PC_	4.2727	1.0000	0.0987	1.0000	−3.2994	0.0005	24.0587	0.0005	I/1
GFC	−1.9393	0.0262	10.4294	0.1077	−7.0152	0.0000	40.8180	0.0000	I/1
TP_M_	−10.9896	0.0000	52.9877	0.0000					I/I

**Table 2:** Co-integration Test results.

Test	Dependent Variable EQ		Dependent Variable FD_EC_		Dependent Variable FD	
	t-Statistic	Probability	t-Statistic	Probability	t-Statistic	Probability
ADF	−1.993263	0.0231	−2.011199	0.0222	−2.562804	0.0052
Residual variance	28341.52		0.561024		0.000578	
HAC variance	55884.49		1.239454		0.000608	

ADF Test in Table two provides the t-statistic value for the Augmented Dickey-Fuller (ADF) test is −1.993263, −2.011199 & −2.562804. The associated probability is 0.0231, 0.0222 & 0.0052. Based on these results, at a conventional significance level of 0.05, we can reject the null hypothesis of no co-integration. This suggests that there is evidence of co-integration among the variables in the model. Residual Variance: The estimated residual variance is 28341.52. HAC Variance: The estimated heteroscedasticity and autocorrelation consistent (HAC) variance is 55884.49. This indicates that the standard errors of the estimated coefficients have been adjusted to account for potential heteroscedasticity and autocorrelation in the data. However, Kao test results are inclined with the study of [55].

After accounting for the endogeneity issue in the time series, Table Three shows that the FMOLS models are subcategories of multiple time series models that directly assess the long-term impact of the independent factors on the dependent variables [56]. Additionally, we knew a co-integrating equation model, FMOLS [57]. first developed the (FM-OLS) regression to offer the best estimates of co-integrating regressions. It is stated from the table that LNCO_2_ is taken as the dependent variable, and the relationship among the variables is significant, which means that the variables are co-integrated among each other. Only the TPM variable signifies insignificance, whereas the rest are substantial. From Table 3, it can be seen that the coefficient of LGE is negative, which means that a 1 % increase in LNCO_2_ decreases LGE by 212 %. Most of these results are identical to those from Ref. [58].

**Table 3:** Panel fully modified least squares (FMOLS) results.

Variable	Dependent Variable EQ			Dependent Variable FD_EC_			Dependent Variable FD		
	Coefficient	t-Statistic	Probability	Coefficient	t-Statistic	Probability	Coefficient	t-Statistic	Probability
LEQ	–	–	–	−0.036573	−9.774562	0.0000∗∗∗	−0.000278	−0.590059	0.5563
LGR	199.7382	4.451986	0.0000∗∗∗	–	–	–	−0.008084	−0.975821	0.3312
LGE	−212.6381	−4.604861	0.0000∗∗∗	–	–	–	−0.002888	−0.387864	0.6988
FDI	3177.323	2.623892	0.0099∗∗∗	−19.21366	−3.545009	0.0006∗∗∗	–	–	–
GDPPC	0.494389	10.9857	0.0000∗∗∗	0.004043	5.456739	0.0000∗∗∗	7.97E-05	−1.817138	0.0718∗
IQI	16.92556	2.912356	0.0043∗∗∗	−0.122917	−7.958436	0.0000∗∗∗	−0.001762	−3.426103	0.0009∗∗∗
TPM	0.139802	0.251066	0.8022	0.052976	12.80243	0.0000∗∗∗	0.001999	5.321585	0.0000∗∗∗

In Model 2, the coefficient of Log Environmental Quality is −0.036573, with a t-statistic of −9.774562, indicating its statistical significance. This negative coefficient suggests that a higher lagged value of environmental quality is associated with decreased fiscal decentralization. This implies environmental degradation may lead to a more centralized fiscal decision-making process. The coefficient of the Financial Development Index is −19.21366, with a t-statistic of −3.545009, indicating its statistical significance. The negative coefficient observed for the financial development index implies that a higher level of economic development is correlated with a decrease in fiscal decentralization. This suggests that a more advanced financial sector may contribute to centralizing fiscal decision-making. The coefficient for the Institutional Quality Index is −0.122917, and its t-statistic is −7.958436, indicating statistical significance. The negative coefficient for the Institutional Quality Index suggests that a higher institutional quality index is associated with decreased fiscal decentralization. It implies that more vital institutions may contribute to centralizing fiscal decision-making. The independent variables are incorporated into the model justification for a significant portion of the variation in the dependent variable, fiscal decentralization. The elevated values of both R-squared and Adjusted R-squared indicate that the model is well-fitted, and the included variables are pertinent in elucidating the variation in fiscal decentralization. The long-run variance, quantified at 2.8234, offers insights into the long-term volatility or variability of the dependent variable, Fiscal Decentralization.

FMOLS methods are subdivisions of multivariate time series forecasting that, after controlling for the endogeneity issues in the time series, directly estimate the protracted consequence of the independent factors on the dependent variables [56]. A co-integrating equation model is another name for FMOLS. In work by Ref. [57], the (FM-OLS) regression was initially developed to offer the best estimates of co-integrating regressions. The table states that carbon emission is taken as the dependent variable, and the relationship among the variables is significant, which means that the variables are co-integrated. Only the TPM variable signifies insignificant, whereas the rest of the variables are essential. Table 3 shows that the coefficient of LGE is negative, which means that a 1 % increase in carbon emission decreases LGE by 212 %. Most of these results are identical to those from Ref. [58].

In model 3, the coefficient of Log Environmental Quality is −0.000278, with a t-statistic of −0.590059 and a probability of 0.5563. This indicates that the coefficient is not statistically significant, suggesting no significant relationship between environmental quality and financial development. The Government Revenue and expenditure coefficient is −0.008084, & −0.002888, with a t-statistic of −0.975821 & −0.387864 and a probability of 0.3312 & 0.6988. Similarly, this coefficient is not statistically significant, suggesting no significant relationship between government revenue and financial development. The coefficient of GDP per Capita is 7.97E-05, with a t-statistic of −1.817138 and a probability of 0.0718. While the coefficient is not statistically significant at conventional levels (p-value >0.05), there is a suggestive sign of a positive bond between GDP per capita and financial development. The coefficient of the Institutional Quality Index is −0.001762, with a t-statistic of −3.426103 and a probability of 0.0009. This statistically significant coefficient indicates that a higher institutional quality index is associated with decreased financial development. The coefficient of the Total Population is 0.001999, with a t-statistic of 5.321585 and a probability of 0.0000. This statistically significant coefficient suggests that a larger population is associated with increased financial development. The long-run variance is 0.001485, which provides information about the long-term volatility or variability of the Financial Development Index. The findings presented in this study closely align with those reported by Ref. [58].

The results from Model One indicate that government revenue, government expenditure, the financial development index, GDP per capita, and the institutional quality index are noteworthy determinants of Environmental Quality (EQ). This underscores the importance of these factors in fostering economic well-being and implies potential policy implications for policymakers seeking to enhance EQ and promote sustainable economic development. Model Two suggests that environmental degradation, increased financial development, more vital institutions, and larger populations may contribute to a more centralized fiscal decision-making process. Conversely, higher GDP per capita is associated with greater fiscal decentralization. These findings offer insights that can be valuable for policymakers when crafting strategies to promote fiscal decentralization based on the specific characteristics and needs of the economy.

Model Three suggests that environmental quality, government revenue, and government expenditure do not significantly impact financial development. This indicates that other factors or dynamics may play a more dominant role in influencing financial development. However, there is suggestive evidence of a positive link between GDP per capita and financial development. Also, more vital institutions and a larger population are associated with higher financial development. However, it is essential to note that the negative R-squared and Adjusted R-squared values indicate that the model does not sufficiently explain the variation in financial development, and further research or alternative models may be needed to understand better the factors driving financial development.

Table four specifically evaluated the weak long-run PPP, which contends that although nominal exchange rates and aggregate price ratios increase in tandem over the long run, they might not be precisely related. The co-integrating slope may, therefore, be near to 1 while being distinct from 1. The table shows that only FDI has a negligible effect on LNCO_2_ [59]. experimentally tested the purchasing power parity (PPP) hypothesis using the group-mean PDOLS estimator. For convenience, the output was condensed into a structured table. To get precise findings, we provided many choices. The "complete" option shows the estimation outcomes for each panel unit. For this empirical application, we replicate Pedroni's original usage of the program and set the DOLS regression's lags and leads to 4 by specifying lags (4) and the Bartlett kernel's lags used in the Newey-West long-run variance of the residuals to 4 by specifying lags (4). Because [60] depended on a broader sample period than the [59] dataset we are presently utilizing, the results will be contradictory with those discovered there. Here, we list the option which mutes the PDOLS estimate findings. The findings show that the aggregate Consumer Price Index ratio and the log of the exchange rate have a co-integrating connection overall. Because all test statistics are distributed N, statistical inference is simple (0,1). All tests are significant, at least at the 10 % level, except the panel t and ADF statistics. The PDOLS findings also provide credence to the weak long-run PPP theory. While many are noticeably higher or lower, most coefficients are near 1. See Ref. [59], (see Table 4).

**Table 4:** Panel dynamic least squares (DOLS) results.

Variable	Dependent Variable EQ			Dependent Variable FD_EC_			Dependent Variable FD		
	Coefficient	t-Statistic	Prob.	Coefficient	t-Statistic	Prob.	Coefficient	t-Statistic	Prob.
LEQ	–	–	–	0.011669	1.352431	0.1810	0.005457	3.573187	0.0008∗∗∗
LGR	193.4509	3.874721	0.0003∗∗∗		–	–	−0.04662	−2.4405	0.0184∗∗∗
LGE	−182.419	−3.51359	0.0009∗∗∗		–	–	0.019877	1.339974	0.1866
FDI	−939.286	−0.95389	0.3444	−14.5757	−2.01916	0.0477∗∗	–	–	–
GDPPC	0.446141	6.001175	0.0000∗∗∗	0.003829	4.736958	0.0000∗∗∗	−0.0004	−4.6203	0.0000∗∗∗
IQI	18.00718	5.826021	0.0000∗∗∗	−0.11535	−6.80865	0.0000∗∗∗	0.003358	2.955809	0.0048∗∗∗
TP_M_	1.953269	3.467437	0.0010∗∗∗	−0.04603	−2.44987	0.0170∗∗∗	0.013708	4.264757	0.0001∗∗∗

Table five, VECM assess the short-run characteristics of the co-integrated series since when co-integration is found between series, we know there is a long-term equilibrium link between them. If there is no co-integration, the VECM is no longer necessary, and we proceed directly to Granger causality tests to determine the causes of the relationships between the variables. The co-integration test shows a long-term stability connection between the two variables, but more testing is required to determine whether there is a causal link. The explanatory power of the regression can be significantly improved if variable A is beneficial in predicting B, i.e., the regression of B is based on previous B values, and past A values are included. Ten A can be referred to as the Granger cause of B if it is not the non-Granger cause. The p-value is below the 5 % threshold for significance, which suggests that the Granger causation must be accepted as the null hypothesis. Table 5 shows that all other relationships have a Granger causal link, except LGDP and LCP, which are not Granger causes of one another. Therefore, oil prices are the Granger cause of GDP and carbon emissions. According to the Granger causality test, there are short-term Granger causal relationships between changes in GDP, carbon emissions, and oil prices and a degree of reciprocity between the three variables. The null hypothesis—that Granger causality exists—must be accepted because it is at a significant level of 5 %. The table reveals that all other connections exhibit a Granger causal link, except for LGDP and LCP, which do not Granger cause each other. Consequently, oil prices emerge as the Granger cause for GDP and carbon emissions. This underscores the short-term Granger causal relationships between changes in GDP, carbon emissions, and oil prices, indicating a degree of reciprocity among these three variables, as noted in the Granger causality test.

**Table 5:** VECM estimation results.

Variables	Dependent Variable (EQ)			Dependent Variable (FD_EC_)			Dependent Variable (FD)		
	Coefficient	t-Statistic	Prob.	Coefficient	t-Statistic	Prob.	Coefficient	t-Statistic	Prob
**GDPPC (-1)**	5.323	0.941	0.3468	2.046	111.546	0.0000∗∗∗	0.000	10.051	0.0000∗∗∗
GDPPC (−2)	−7.534	−1.489	0.0369	−1.046	−56.275	0.0000∗∗∗	0.000	−10.054	0.0000∗∗∗
LEQ (−1)	−230.111	−0.952	0.3416	−994061	−1.201	0.2301	−1.048	−1.215	0.0249∗∗
LEQ (−2)	277.979	1.193	0.0332∗∗	500640	0.620	0.5358	1.049	1.157	0.2479
LGR (−1)	0.287	3.506	0.0005∗∗∗	436.510	1.523	0.0283∗∗	−26.011	−1.710	0.0878∗
LGR (−2)	−0.209	−2.356	0.0187∗∗	−374.971	−1.207	0.2279	30.204	2.058	0.0400∗∗
LGE (−1)	−1.861	−0.105	0.9164	−147239.	−2.390	0.0171∗∗	481.400	0.397	0.6914
LGE (−2)	−8.370	−0.486	0.6272	138713	2.306	0.0214∗∗	685.635	1.031	0.0930∗
FDI (−1)	482.714	0.982	0.0263∗∗	2362219	1.387	0.0658	−417.649	−0.629	0.5293
FDI (−2)	19.575	0.040	0.9683	−2072934	−1.220	0.2228	1.318	11.766	0.0000∗∗∗
FD (−1)	0.986	8.557	0.0000∗∗∗	−622.317	−1.554	0.1205	−0.281	−2.316	0.0208∗∗
FD (−2)	0.652	0.320	0.9864	678.056	1.611	0.0976∗	−20.460	−0.850	0.3955
IQI (−1)	−0.401	−0.204	0.7491	8425.493	1.194	0.2330	15.747	0.670	0.5029
IQI (−2)	0.551	0.104	0.8388	−20721	−3.042	0.0024∗∗∗	89.220	0.276	0.7827
TQM (−1)	−0.738	−0.137	0.9173	658390	1.170	0.2422	72.959	0.231	0.8173
TQM (−2)	−2.781	−0.017	0.8909	0	1.550	0.0815∗	0.000	−2.539	0.0113∗∗
R-squared	0.646408			0.839506			0.922842		
Adjusted R-squared	0.589834			0.817478			0.912252		
S.E. of regression	1.91586			0.049805			0.678937		
Durbin-Watson stat	1.980482			2.037512			1.881002		

## 5 Conclusion

Our comprehensive analysis of the unified relationships among financial development, environmental quality, and fiscal decentralization in densely populated developing countries such as China, India, and Pakistan offers a unique perspective on the escalating challenges associated with global warming. Employing a substantial panel dataset and robust econometric methods, we delineate how diverse policies yield distinct effects across various national contexts, revealing intricate temporal interdependencies within this system. VECM, DOLS, and FMOLS models have offered a nuanced understanding of the intricate relationships among environmental quality, fiscal decentralization, and financial development. The results highlight the positive short-term influences of GDP per capita and government revenue on environmental quality, while fiscal decentralization, government expenditure, and institutional factors exhibit varied impacts. Financial development demonstrates both positive and negative associations with different variables, underscoring the complexity of its relationship with fiscal decentralization and government actions. The collective models stress the need for customized policy interventions that consider the diverse impacts of various factors on sustainable development. Policymakers can use these insights to design strategies for the multifaceted dynamics influencing environmental, fiscal, and financial trajectories, promoting a more sustainable and resilient development path. This research contributes valuable knowledge for policymakers seeking informed approaches to navigate the complex terrain of sustainable development.

This study underscores the significance of accounting for cross-sectional interdependence and acknowledging the diverse paths taken by different economies. The varied results emphasize the necessity for customized policy interventions, moving beyond one-size-fits-all approaches and adopting strategies tailored to specific situations. Policymakers can leverage our findings to formulate interventions that maximize the benefits of decentralization while mitigating potential drawbacks, ensuring that financial development serves as a catalyst for environmental preservation rather than a contributor to future degradation. By unraveling the complexities of the tripartite interaction among fiscal decentralization, financial development, and environmental quality within highly populated countries, this research significantly contributes to the ongoing battle against global warming. It furnishes valuable insights that can inform evidence-based policymaking, guide researchers in focused investigations, and assist policymakers in crafting practical solutions to address the escalating environmental challenges facing our world. A nuanced understanding of these processes paves the way for a future where growth and ecological sustainability coexist harmoniously as we navigate the interconnected complexity of these systems.

Future research should explore the impact of institutional quality, governance, and political stability on the relationship between fiscal decentralization, financial development, and environmental quality. A deeper understanding of how these elements interact with intergovernmental relations and environmental policies can offer valuable insights for enhancing policy effectiveness. Additionally, examining the effects of shocks to intergovernmental fiscal relations—such as those caused by financial crises or natural disasters—on the dynamics between fiscal decentralization, financial development, and environmental quality could provide important insights into the resilience of various government policy frameworks.

Policymakers need to consider the unique financial, social, and environmental circumstances of their intergovernmental relationships when crafting financial and environmental policies. Approaches tailored to local conditions are more likely to succeed in advancing sustainable development. Enhancing institutional quality and governance is vital for the successful implementation of these policies. By improving transparency, accountability, and regulatory frameworks, the positive effects of these policies on environmental quality can be amplified.

## CRediT authorship contribution statement

**Yi Ding:** Supervision, Resources, Project administration, Methodology. **Fayaz Hussain Tunio:** Writing – original draft, Supervision, Formal analysis, Data curation, Conceptualization. **Agha Amad Nabi:** Writing – review & editing, Software, Resources, Methodology, Formal analysis.

## Declaration of competing interest

The authors declare that they have no known competing financial interests or personal relationships that could have appeared to influence the work reported in this paper.
